# Systemic Sclerosis Is Linked to Psoriasis and May Impact on Patients’ Survival: A Large Cohort Study

**DOI:** 10.3390/jcm8040521

**Published:** 2019-04-16

**Authors:** Abdulla Watad, Nicola Luigi Bragazzi, Dennis McGonagle, Giovanni Damiani, Doron Comaneshter, Arnon Cohen, Howard Amital

**Affiliations:** 1 Department of Medicine B and Zabludowicz Center for Autoimmune Diseases, Sheba Medical Center, Tel-Hashomer, Ramat-Gan 5265601, Israel; howard.amital@sheba.health.gov.il; 2 Sackler Faculty of Medicine, Tel-Aviv University, Tel-Aviv 6997801, Israel; 3 Section of Musculoskeletal Disease, Leeds Institute of Molecular Medicine, University of Leeds, NIHR Leeds Musculoskeletal Biomedical Research Unit, Chapel Allerton Hospital, Leeds LS7 4SA, UK; D.G.McGonagle@leeds.ac.uk; 4 Postgraduate School of Public Health, Department of Health Sciences (DISSAL), University of Genoa, 16132 Genoa, Italy; 5 Department of Dermatology, Case Western Reserve University, Cleveland, OH 44124, USA; dr.giovanni.damiani@gmail.com; 6 Young Dermatologists Italian Network, Centro Studi GISED, 24122 Bergamo, Italy; 7 Chief Physician’s Office, Clalit Health Services, Tel-Aviv 6997801, Israel; doronko1@clalit.org.il; 8 Siaal Research Center for Family Medicine and Primary Care, Faculty of Health Sciences, Ben Gurion University of the Negev, Beer Sheva 84101, Israel; arcohen@clalit.org.il

**Keywords:** psoriasis, systemic sclerosis, scleroderma, big data, massive data mining, nation-wide survey

## Abstract

Although skin manifestations are quite common in systemic sclerosis (SSc), a link between SSc and psoriasis (PsO) has been poorly investigated. We assessed the Clalit medical database in a cohort study to compare the prevalence of PsO between SSc-patients and SSc-free controls. We also evaluated the SSc-related autoantibodies’ role in the co-existence of the two conditions. Survival analysis was performed using both univariate (Kaplan–Meier, log-rank test) and multivariate (Cox proportional-hazards technique) analyses. Our cohort of 2,431 SSc-patients was age- and gender-matched with 12,710 controls (case-control match 1:5.2). There were 150 (1.2%) cases of PsO among controls and 47 (1.9%) among SSc-patients (*p* = 0.0027). A SSc diagnosis was an independent risk factor for PsO with an odds ratio (OR) of 2.16 (95%CI 1.38–3.39, *p* = 0.0008). Among SSc-patients, 98.6% with PsO were antinuclear antibodies (ANA)-negative. In terms of survival, the mortality rate in SSc-patients with PsO was lower than SSc without PsO (14.9% vs. 26%, *p* < 0.0001). At the multivariate-analysis, SSc-patients with PsO compared to SSc-patients without PsO had an OR for death of 0.44 (95%CI 0.19–0.99, *p* < 0.05). SSc is independently associated with PsO. The cases with concurrent PsO and SSc are almost exclusively ANA-negative and may exhibit a better survival.

## 1. Introduction

Systemic sclerosis (SSc) is an autoimmune disorder that involves both connective tissue and skin by damaging vessels architecture and, finally, leading to fibrosis [1]. Remarkably, SSc has the highest disease-related mortality and substantial non-lethal complications among rheumatic diseases [2]. 

Although clinical signs and symptoms such as Raynaud’s phenomenon are prototypical of SSc, this disease globally exhibits a *plethora* of clinical manifestations with a high inter-individual variability [3]. 

In SSc patients, skin assessment has a pivotal role because of its thickening and is a well-known marker of internal organ involvement, and of poor prognosis [4,5]. Other skin manifestations are classically described in the context of SSc and include digital ulcers, calcinosis, puffy fingers, and telangiectasia [5,6]. Immunopathologically, the presence of autoantibodies and major histocompatibility complex type-II (MHC-II) associations strongly support the role of classical humoral autoimmunity in SSc disease pathogenesis [7,8].

Psoriasis (PsO) is a chronic, complex, multi-factorial, systemic inflammatory disease [9,10] that merges some aspects belonging to autoimmunity and autoinflammation [11]; it clinically manifests as erythematous, variously infiltrated, large edges squamous plaques with a prevalence rate of 2–3% worldwide [12]. 

Unlike the classical humoral mediated autoimmune diseases, at the population level, PsO has a different genetic architecture with strong major histocompatibility type-I (MHC-I) associations, especially HLA-Cw6, ERAP-1 and other single nucleotide polymorphisms (SNPs) pointing to CD8+ T-cell related immunopathology [8,13]. Experimental and clinical evidence suggest also a leading role for autoreactive T-cells [14,15,16], however the autoantigen is still elusive and the only pathogenetic mechanism fully described remains the LL-37-self DNA driven [17]. Several other autoantigens were proposed [18], however this topic is still debated.

The co-occurrence of SSc and PsO has been rarely reported and interestingly, such cases were generally antinuclear antibodies (ANA) negative [19,20]. Furthermore, autoimmune mechanisms play a major role, especially the convergence on the type-I interferons pathway SNPs in SSc [18,21] and on different type-I interferon pathway SNPs in PsO [22]. 

Thus, we carried out a nationwide epidemiological survey in order to assess the possible link between PsO and SSc, and to enlighten the typical characteristics of this rare subgroup of patients, namely prevalence, survival, prognosis, and the role of SSc-linked autoantibodies.

## 2. Experimental Section

### 2.1. Ethical Clearance

The present study protocol was reviewed and received ethical clearance by the Ethical Committee of the Clalit Health Services (CHS), based at the Soroka Medical Center, Beer-Sheva, Israel (ethical code 0212-17-COM2).

### 2.2. Design, Sample and Procedures

CHS represents the major Israeli health organization, delivering both public and semi-private healthcare services to about 4,400,000 insured subjects (approximately half of the entire Israeli population). The chronic disease CHS registry collects data from different sources, including pharmaceutical, medical and administrative ones.

Being an electronic registry, highly computerized and continuously updated (from 2000 to 2017), CHS enables researchers to automatically retrieve and extract relevant patient data performing extensive nation-wide, real-time epidemiological surveys *via* massive data-mining. CHS was mined to obtain a cohort of SSc patients, properly age- and sex-matched with controls, which were SSc-free and were randomly selected with a case-control match of 1:5.2.

The present study was devised as a cohort study, with further sub-analyses conducted among cases to shed light on predictors of PsO among SSc patients.

### 2.3. Measures

Definition of SSc or PsO and consequently patient classification and categorization were based on a clearly documented diagnosis, present at least twice in the medical records registered by a community physician or in the hospital discharge letter form, signed by a specialist. All SSc patients in the registry meeting with these criteria were eligible and were, therefore, enrolled in the present study.

Available data included age, sex, socioeconomic status (SES), body mass index (BMI) and diagnoses of co-morbidities (in particular, chronic diseases). Specifically, SES was computed based on the poverty index of the insured subject’s residence area, using relevant information from the 2008 National Census. In order to capture the nonlinear relation between BMI and dependent variables, BMI was categorized in underweight (<20 kg/m^2^), normal weight (20–25 kg/m^2^), overweight (25–30 kg/m^2^), and obese (>30 kg/m^2^). Normal weight was used as a reference.

Previously studies extensively demonstrated the high validity and reliability of the diagnoses in the registry [23,24,25,26,27]. Serum samples of SSc-patients are routinely drawn, collected and assessed. In the present investigation, a panel comprising of the following autoantibodies was analyzed: antinuclear (ANA), anti-centromere, anti-Scl-70 (topoisomerase-I), anti-RNA polymerase III and anti-ribonucleoprotein (anti-RNP). A positive test was defined as supplied by the kit assay insert and manufacturer’s instructions. In case of multiple/serial assessment of autoantibodies at different time-points during the study period, patients were considered positive for an autoantibody if they were ever positive based on clinically obtained assays.

### 2.4. Statistical Analyses

Categorical socio-demographic and clinical variables, including PsO rates were compared between cases and controls utilizing the chi-squared test, whereas the Student’s *t*-test and one-way analysis of variance (ANOVA) were carried out for continuous parameters (between two and more groups, respectively). 

In case of violation of the normality of data distribution, non-parametric versions of the above-mentioned tests were preferred.

The link between SSc and PsO was assessed by performing a classical unmatched multivariate logistic regression analysis, adjusting for possible confounders. Unmatched models were used instead of matched regression techniques, since the matching of the present study between cases and controls was loose (that is to say, based on a small number of parameters, such as age and gender).

Dates of registration in the medical records of SSc or the start of follow-up for controls, PsO and death, anthropometric information and medical co-morbidities, were retrieved and downloaded from the database.

Survival analysis was performed using both univariate (Kaplan–Meier curves, log-rank test) and multivariate (Cox proportional-hazards technique) analyses to detect covariates significantly conferring an increased risk of all-cause mortality.

Missing data were handled by performing multiple imputation analysis.

All statistical analyses were performed with the commercial software “Statistical Package for Social Sciences” for Windows (SPSS version 24.0, IBM, Armonk, NY, USA). For all analyses, significance threshold was set at 0.05.

## 3. Results

### 3.1. Basic Characteristics of the Study Population

**Figure 1:**
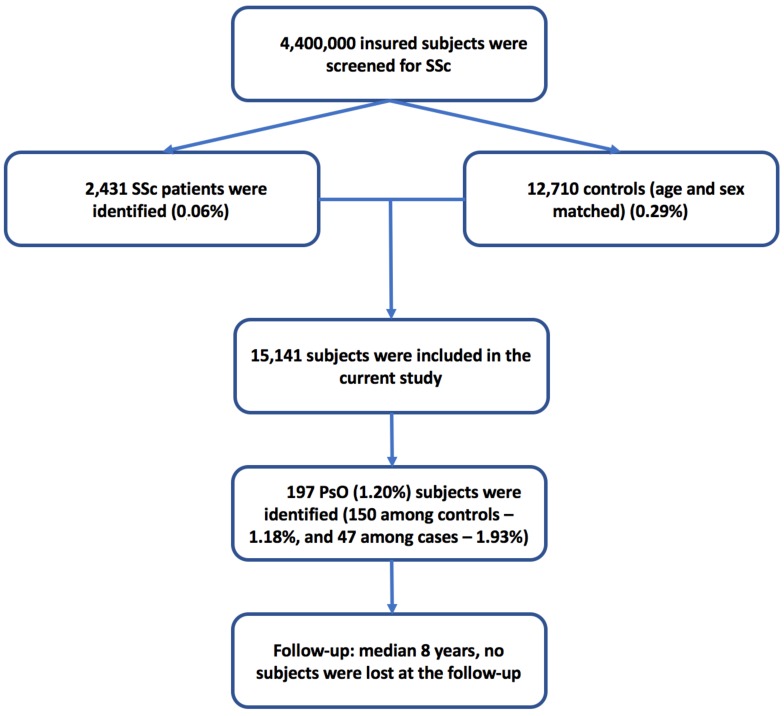
Flowchart of the algorithm of patient selection and inclusion. SSc: systemic sclerosis, PsO: psoriasis.

The process of patient selection and inclusion is pictorially shown in Figure 1.

The study sample comprised of 15,141 subjects aged 63.32 ± 18.06 years (median 66 years), 2764 males and 12,377 females (with a female/male ratio of 1:4.5). There were 2431 SSc patients matched with 12,710 controls (case-control match 1:5.2) (Table 1).

Approximately, 39.3% of SSc cases suffered from gastroesophageal reflux disease (GERD) and 19.4% from pulmonary hypertension, data in line with prevalence rates reported in literature and, as such, further corroborating the diagnosis of SSc. Moreover, both GERD and pulmonary hypertension were associated with SSc diagnosis by the chi-squared test (*p* < 0.0001, in both cases).

Being age- and gender-matched, no differences were found between cases and controls concerning mean age and gender. The two groups also did not differ in terms of smoking habit. On the contrary, BMI (*p* < 0.0001) and SES (*p* < 0.0001) differed between SSc patients and controls. Overweight and obese subjects were less represented among SSc, as well as subjects from medium and high SES with respect to low SES. Table 1, shows the overall population, SSc patients and age-and-sex matched controls—basic characteristics.

Concerning PsO as co-morbidity, 197 (1.3%) cases of PsO were diagnosed: 150 (1.2%) among controls and 47 (1.9%) among SSc patients (*p* = 0.0027). The mean age of PsO onset in SSc and those without SSc was 56.09 ± 16.25 and 58.90 ± 14.39 years, respectively (*p* = 0.0740) (Table 2). Further details are reported in Table 2.

### 3.2. Mortality Rates and Survival Analysis

**Figure 2:**
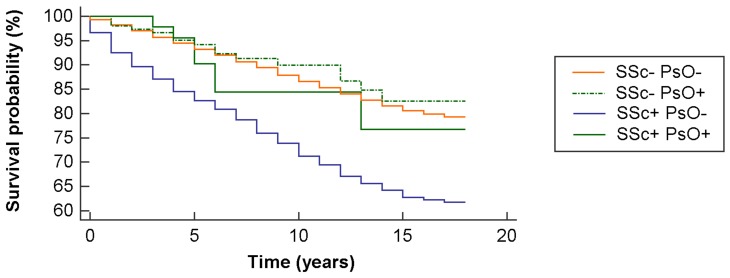
Survival probability according to the Kaplan-Meier analysis, stratified based on the occurrence of systemic sclerosis (SSc) and psoriasis (PsO) diagnosis.

At the survival analysis, the log-rank test achieved the threshold of statistical significance (chi-squared = 357.67, degrees of freedom = 3, *p* < 0.0001) (Figure 2). The overall mortality rate was 14.7% with 2226 deaths, 12.5% among controls (1,589 deaths) and 26.2% among cases (637 cases). As expected, the mortality rate was significant in SSc compared to controls (*p* < 0.0001). Interestingly, the mortality rate in SSc patients with PsO was lower than SSc patients without PsO (14.9% vs. 26%, *p* < 0.0001 after adjusting according to Bonferroni for multiple testing) (Table 2). At the multivariate analysis, in terms of risk of death, SSc patients with PsO in comparison to SSc patients without PsO had an odds ratio (OR) of 0.44 (95%CI 0.19–0.99, *p* = 0.05).

### 3.3. Independent Predictors of Psoriasis

We found that SSc was significantly associated with PsO (OR 2.16, with a range of 1.38–3.39, *p* = 0.0008). In keeping with the literature [23,24,25,26,27], at the multivariate logistic regression (Table 3), covariates statistically associated with PsO were a BMI greater than 30 kg/m^2^ (OR 2.32, with a range of 95%CI 1.14–4.73, *p* = 0.0202), medium and high SES (OR 1.65, with a range of 1.14–2.41, *p* = 0.0086, and OR 1.90, with a range of 95%CI 1.25–2.89, *p* = 0.0029, respectively), and smoking (OR 1.50, with a range of 1.08–2.08, *p* = 0.0155).

### 3.4. Independent Predictors of All-Cause Mortality

Among SSc cases, 67.9% were tested for at least one autoantibody: in more detail, 78.7% were tested for ANA, whereas 61.1% for anti-Scl-70, 49.9% for anti-centromere, 41.5% for anti-RNP and 10.0% for anti-RNAPIII. Among subjects tested, 84.1% resulted ANA positive, while 39.4% were positive for anti-Scl-70, 32.0% for anti-centromere autoantibody, with 15.0% and 3.3% being anti-RNAPIII and anti-RNP positive, respectively. Double positivity at any time of study period was found to be low.

At the Cox multivariate survival analysis, covariates found to be statistically associated with all-cause mortality were age (HR 1.06, in the range 95%CI 1.06–1.07, *p* < 0.0001), BMI (in the range 20–25 kg/m^2^ HR 0.74, in the range 95%CI 0.62–0.87, *p* = 0.0002; in the range 25–30 kg/m^2^ HR 0.58, in the range 95%CI 0.49–0.69, *p* < 0.0001; greater than 30 kg/m^2^ HR 0.69 95%CI 0.59–0.82, *p* < 0.0001). sex (female gender, HR 0.73, in the range 95%CI 0.65–0.83, *p* < 0.0001), high SES (HR 0.71, in the range 95%CI 0.62–0.82, *p* < 0.0001) and smoking (HR 0.88, in the range 95%CI 0.79–0.99, *p* = 0.0287). Interestingly, SSc (HR 2.36, in the range 95%CI 2.07–2.70, *p* < 0.0001), but not the matched PsO cohort (HR 0.67, in the range 95%CI 0.41–1.10, *p* = 0.1137), was associated with all-cause mortality.

### 3.5. Role of SSc-Linked Autoantibodies in the Co-Existence of SSc and PsO

At the multivariate logistic regression, the risk of PsO in SSc patients was inversely associated with positivity for ANA auto-antibody with an OR of 0.43 (95%CI 0.21–0.89, *p* = 0.0239), after adjusting for age, gender, BMI, smoking status and SES (see Table 4). In other words, 98.4% of SSc patients with PsO were ANA negative.

### 3.6. The Temporal Relationship between SSc and PsO Diagnoses

To explore this further, we looked at the temporal onset of both conditions. Whilst 11.9% of subjects were diagnosed with SSc and PsO within a year of each other, 52.4% were diagnosed firstly with PsO and after a mean time of 7.09 ± 4.86 years were diagnosed with SSc. By contrast, SSc was diagnosed in 35.7% of subjects at a mean time of 6.04 ± 4.49 years before PsO.

At the multivariate Cox proportional-hazards model, the risk of developing PsO among SSc cases was computed to be 2.36 ([95%CI 1.51–3.69], *p* = 0.0002).

## 4. Discussion

This is the first study to address the link between SSc and PsO and to further explore the impact of their co-existence on patient survival. Our study showed that SSc is independently associated with PsO, almost exclusively in those ANA negative and such coexistence may have a patient survival benefit.

Generally speaking, patients with an autoimmune disease are at a higher risk for another autoimmune disorder and this can be attributed to several factors including dysregulated immune pathways in the host [28], shared environmental risk factors [29,30], genetic predisposition with specific HLA profiles [31], and other epigenetic factors. However, at the population level, SSc and PsO have fundamentally different immunopathogenetic pathways [32,33]. Thus, given the ANA negative status in the SSc cases with PsO it is tempting to speculate a skin specific convergence of type-I IFN and CD8+ T-cell related pathology in this setting [34].

An anecdotal association between the co-existence of SSc and PsO was previously reported in only four subjects. Harrison et al. [19] described three cases with concurrent SSc and PsO with a precise temporal relationship between the development of both. Furthermore, in these three patients, SSc developed very aggressively, with a rapidly progressive skin involvement. In all three cases, patients developed SSc after being already diagnosed with PsO. Another case reported the development of Raynaud’s phenomenon, sclerodactyly and a diagnosis of SSc in a 61-year-old woman, with a two-year history of PsO and psoriatic arthritis [20]. ANA, anti-DNA, and anti-ENA were negative.

Remarkably, Kuo and collaborators [35] have conducted a large study to estimate the risk for autoimmune diseases among first degree relative of SSc patients. They found that the adjusted relative risk for PsO in first-degree relatives of SSc patients was 1.52 (95%CI 1.15–2.01).

In the two studies that previously reported the 4 patients with both SSc and PsO diagnosis, 3 of 4 were ANA negative which is similar to our study with a predominant ANA negativity (nearly 99%). Even thought it could be expected that SSc patients with PsO may have an immunological profile characterized by ANA positivity, the vast majority of existing studies did not assess the immunological profile.

Several hypothesis or mechanisms can be postulated as an explanation for a putative link between SSc and PsO. With regard to the shared genetic background, associations between MHC-I molecules such as increased frequency of HLA-B*08:01 in SSc [36] and PsO, mainly psoriatic arthritis [37] have been reported. However, a large genome-wide association (GWAS) study did not specifically report on this association in SSc [8]. 

Furthermore, both diseases share some immunopathogenic aspects. In PsO, skin inflammation is dominated by CD4+ and CD8+ T-cells [38] but the CD8+ T-cells are emerging as key players [39]. In SSc, CD4+ T-cells including follicular helper T-cells may drive autoantibody production and interactions between CD4+Th2 T-cells and fibroblasts releasing profibrotic mediators (IL-4, IL-6, IL-13) may contribute to the skin phenotype [40]. Type-I interferon pathways play an important role in both diseases and studies have described the development of both conditions in subjects submitted to intense interferon therapy in subjects treated for hepatitis C infection, multiple sclerosis and myeloproliferative diseases [41,42].

An important plausible mechanism linking SSc and PsO is the skin target tissue damage (including radiation and direct trauma) leading to the exposure of neo-autoantigens to the immune system that may trigger local adaptive immune reactions [43,44]. Indeed, it has been reported that autoreactive T-cells in the skin behave very differently depending on their local inflammatory environment [45]. Therefore, a barrier dysfunction in environmentally exposed organs such as the skin, and aberrant innate immune reactions can often trigger secondary adaptive immune CD8+ T-cell responses that may be the mechanism behind the co-occurrence of the two conditions [46].

Interestingly, PsO patients have a high level of LL-37 that in some circumstances binds also self-DNA, triggering plasmacytoid dendritic cell to produce type-I IFN that leads to self-antigen presentation and autorective T cells [17]. Moreover, UV therapy can induce keratinocytes apoptosis with nuclear antigen redistribution during apoptotic bleb formation providing nuclear autoantigens leading to autoimmune reactions [47]. Finally, skin structural damage and keratinocytes apoptosis with barrier dysfunction may enable the entrance of different adjuvants such as infectious agents that lead to the activation of different immune pathways including innate and adaptive immunity triggering the development of SSc. However, this still needs to be established.

A novel finding of our study is that SSc patients with PsO had a significantly better survival rate than those SSc patients without PsO. This may support the theory that local inflammation induces local autoantigen modifications and therefore the autoimmune reactions induced exclusively in the skin, rather than a full-blown systemic disease with internal organ involvement and its consequent survival reduction. On the other hand, this may reflect that SSc in those patients with PsO is a distinct disease entity, diverging in respect of serological profile, mortality rates, and probably even immunopathologically.

Our study has several strengths, being based on a representative computerized database of 4.4 million enrolled subjects over 17 years, and being less prone to selection and ascertainment biases that plague single-centre studies. Moreover, we utilized one of the largest cohorts of SSc patients. However, our study suffers from some drawbacks that should be properly recognized. The major limitation is that we could not differentiate among the different clinical variants of either SSc (like localized and systemic, which can be further subdivided into diffuse and limited variants) or PsO (such as plaque, guttate, inverse, pustular, erythrodermic or other variants). Further, we had no data concerning the pharmacological treatment administered to the patients.

However, from our previous report [48], only 3.8% of PsO-patients received systemic therapy, which, on the other hand, fails to explain the extremely high percentage of ANA negativity in patients with both conditions. Based on the above-mentioned shortcomings, further research could explore the different clinical forms of SSc and PsO and the impact of pharmacological treatment.

## 5. Conclusions

In conclusion, a significant association between SSc and PsO and matched controls was found in the present nation-wide survey. ANA negativity seems to play a protective role in the co-existence of both conditions being linked to a better prognosis. Several mechanisms could explain this link including the shared immunopathogenetic mechanisms in a rare subgroup or the potential impact of local or systemic therapies in precipitating or modification of the course of the associated skin disease.

Remarkably, this study has described a subgroup of SSc patients with a particular immunological profile that seemed to confer a better prognosis in terms of survival. This suggests the need of adopting an individualized approach rather than “one-size-fits-it-all”. Even though considering inter-individual clinical differences and inscribing them in the broader and more complex picture of SSc is of crucial importance, replication and validation of this rare disease subgroup might be important for mechanistic disease classification of SSc, therapy stratification and prognostication.

## Figures and Tables

**Table 1:** Main characteristics of cases (systemic sclerosis (SSc)-patients) and controls recruited.

Characteristic	All Population (n = 15,141)	Controls without SSc (n = 12,710)	SSc Patients (n = 2431)	Statistical Significance (*p*-Value)
Age (mean ± SD; median)	63.32 ± 18.06; 66	63.44 ± 18.08; 66	62.69 ± 17.90; 66	NS
Age at diagnosis/beginning of the follow-up (mean ± SD; median)	54.57 ± 18.64; 57	54.54 ± 18.63; 57	54.77 ± 18.67; 57	NS
Gender (female; %)	12,377 (81.7%)	10,390 (81.7%)	1987 (81.7%)	NS
BMI (n; %) ^a^				<0.001
<20 kg/m^2^	1283 (9.2%)	1098 (8.6%)	185 (15.6%)	
20–25 kg/m^2^	4189 (30.1%)	3803 (29.9%)	386 (32.5%)	
25–30 kg/m^2^	4380 (31.5%)	4055 (31.9%)	325 (27.4%)	
>30 kg/m^2^	4045 (29.1%)	3754 (29.5%)	291 (24.5%)	
SES (n; %) ^b^				<0.001
Low	5763 (40.4%)	4769 (39.7%)	994 (44.4%)	
Medium	5364 (37.6%)	4543 (37.8%)	821 (36.7%)	
High	3122 (22.0%)	2699 (22.5%)	423 (18.9%)	
Smoking (n; %)	4332 (28.6%)	3628 (28.5%)	704 (29.0%)	NS
Psoriasis (n; %)	197 (1.3%)	150 (1.2%)	47 (1.9%)	0.0027
All-cause mortality (n; %)	2226 (14.7%)	1589 (12.5%)	637 (26.2%)	<0.001

^a^ Available for 91.8% of data; ^b^ available for 94.1% of data. Abbreviations: SSc: systemic sclerosis, BMI: body mass index, SES: socioeconomic status, NS: not significant.

**Table 2:** Comparison between systemic sclerosis patients and controls with and without psoriasis (univariate analysis).

Parameter	SSc- PsO-	SSc- PsO+	SSc+ PsO-	SSc+ PsO+	Statistical Significance (*p*-Value)
	(n = 12,560)	(n = 150)	(n = 2384)	(n = 47)	
Age	63.39 ± 18.12	67.76 ± 14.11	62.65 ± 17.96	64.47 ± 14.57	0.0109
Gender (female)	10,268 (81.8%)	122 (81.3%)	1949 (81.8%)	38 (80.9%)	0.9977
BMI					*p* < 0.0001
BMI <20 kg/m^2^	1091 (8.7%)	7 (4.7%)	182 (15.7%)	3 (11.1%)	
BMI 20–25 kg/m^2^	3772 (30.0%)	31 (20.7%)	376 (32.4%)	10 (37.0%)	
BMI 25–30 kg/m^2^	4005 (31.9%)	50 (33.3%)	321 (27.7%)	4 (14.8%)	
BMI >30 kg/m^2^	3692 (29.4%)	62 (41.3%)	281 (24.2%)	10 (37.0%)	
SES					*p* < 0.0001
Low	4731 (39.9%)	38 (27.1%)	978 (44.5%)	16 (39.0%)	
Medium	4478 (37.7%)	65 (46.4%)	807 (36.7%)	14 (34.1%)	
High	2662 (22.4%)	37 (26.4%)	412 (18.8%)	11 (26.8%)	
Smoking status	3573 (28.4%)	55 (36.7%)	680 (28.5%)	24 (51.1%)	0.0009
Mean age at psoriasis onset/beginning of the follow-up	54.48 ± 18.67	58.90 ± 14.39	54.74 ± 18.71	56.09 ± 16.25	0.0740
Mortality rate	1573 (12.5%)	16 (10.7%)	630 (26.4%)	7 (14.9%)	*p* < 0.0001

Abbreviations: SSc: systemic sclerosis, PsO: psoriasis, BMI: body mass index, SES: socioeconomic status.

**Table 3:** Independent predictors for psoriasis (multivariate logistic regression).

Variable	Coefficient	Standard Error	Wald	*p*-Value	OR	95% CI
Age	0.01	0.01	2.47	0.1162	1.01	1.00 to 1.02
BMI 20–25 kg/m^2^	0.21	0.37	0.31	0.5794	1.23	0.59 to 2.56
BMI 25–30 kg/m^2^	0.49	0.37	1.78	0.1823	1.63	0.79 to 3.35
BMI >30 kg/m^2^	0.84	0.36	5.40	0.0202	2.32	1.14 to 4.73
Sex (female)	0.15	0.22	0.50	0.4785	1.17	0.76 to 1.79
SSc	0.77	0.23	11.19	0.0008	2.16	1.38 to 3.39
SES (medium)	0.50	0.19	6.90	0.0086	1.65	1.14 to 2.41
SES (high)	0.64	0.21	8.90	0.0029	1.90	1.25 to 2.89
Smoking	0.40	0.17	5.86	0.0155	1.50	1.08 to 2.08

Abbreviations: SSc: systemic sclerosis, BMI: body mass index, SES: socioeconomic status, OR: odds ratio.

**Table 4:** The risk of psoriasis in SSc patients according to the positivity of auto-antibodies (multivariate logistic regression with adjustment for age, gender, body mass index, smoking status and socioeconomic status).

Variable	Coefficient	Standard Error	Wald	*p*-Value	OR	95% CI
ANA	−0.84	0.37	5.10	0.0239	0.43	0.21 to 0.89
Anti-centromere	0.000	0.35	0.00	0.9992	1.00	0.50 to 2.00
Anti-SCL-70	0.48	0.39	1.47	0.2258	1.61	0.75 to 3.47
Anti-RNA polymerase III	0.72	0.59	1.50	0.2210	2.06	0.65 to 6.59
Anti-RNP	−0.26	1.04	0.06	0.8022	0.77	0.10 to 5.95

Abbreviations: SSc: systemic sclerosis, BMI: body mass index, SES: socioeconomic status, ANA: antinuclear antibodies.
